# Preventive Digital Mental Health for Children in Primary Schools: Acceptability and Feasibility Study

**DOI:** 10.2196/30668

**Published:** 2021-12-13

**Authors:** Sian M Davies, Jenni Jardine, Kerry Gutridge, Zara Bernard, Stephen Park, Tom Dawson, Kathryn M Abel, Pauline Whelan

**Affiliations:** 1 GM.Digital Research Unit, Centre for Women's Mental Health Division of Psychology and Mental Health, School of Health Sciences Faculty of Biology, Medicine and Health, The University of Manchester Manchester United Kingdom; 2 Greater Manchester Mental Health NHS Foundation Trust Manchester Academic Health Science Centre Manchester United Kingdom; 3 Lexplore Ltd Marple United Kingdom; 4 GM.Digital Research Unit, Centre for Health Informatics Division of Informatics, Imaging and Data Sciences The University of Manchester Manchester United Kingdom

**Keywords:** digital mental health, acceptability, feasibility, child and adolescent mental health and well-being, school-based mental health care, prevention, digital assessment and monitoring, reading screening or ability

## Abstract

**Background:**

The incidence of mental health problems in children and adolescents in the United Kingdom has significantly increased in recent years, and more people are in contact with mental health services in Greater Manchester than in other parts of the country. Children and young people spend most of their time at school and with teachers. Therefore, schools and other educational settings may be ideal environments in which to identify those experiencing or those at the risk of developing psychological symptoms and provide timely support for children most at risk of mental health or related problems.

**Objective:**

This study aims to test the feasibility of embedding a low-cost, scalable, and innovative digital mental health intervention in schools in the Greater Manchester area.

**Methods:**

Two components of a 6-week digital intervention were implemented in a primary school in Greater Manchester: Lexplore, a reading assessment using eye-tracking technology to assess reading ability and detect early atypicality, and Lincus, a digital support and well-being monitoring platform.

**Results:**

Of the 115 children approached, 34 (29.6%) consented and took part; of these 34 children, all 34 (100%) completed the baseline Lexplore assessment, and 30 (88%) completed the follow-up. In addition, most children were classified by Lincus as *regular* (≥1 per week) survey users. Overall, the teaching staff and children found both components of the digital intervention engaging, usable, feasible, and acceptable. Despite the widespread enthusiasm and recognition of the potential added value from staff, we met significant implementation barriers.

**Conclusions:**

This study explored the acceptability and feasibility of a digital mental health intervention for schoolchildren. Further work is needed to evaluate the effectiveness of the digital intervention and to understand whether the assessment of reading atypicality using Lexplore can identify those who require additional help and whether they can also be supported by Lincus. This study provides high-quality pilot data and highlights the potential benefits of implementing digital assessment and mental health support tools in a primary school setting.

## Introduction

### Background

The prevalence of poor mental health in children and adolescents has significantly increased in the United Kingdom and, in recent years, has become a public health concern worldwide [1,2]. Greater Manchester has long been identified as an area of high unmet mental health need, with more people in contact with mental health services than in many other parts of the United Kingdom [3]. Common mental disorders in children and adolescents aged 5 to 14 years have been established as a leading cause of disability [4], with 50% of all adult mental health–related problems being diagnosed before the age of 14 years [5]. The rates of probable mental disorders in children and young people (CYP) have steadily and consistently risen, with 1 in 9 children aged 5 to 16 years being identified as having a probable mental disorder [6], which increased to 1 in 6 more recently [7].

The extensive literature has identified links between children’s mental health, academic performance, and outcomes [8-11]. Many factors associated with poor mental health, such as social deprivation [12], are linked with poorer reading ability [13]. Similarly, there is an association between poorer reading ability and developmental and behavioral disorders that develop during childhood and adolescence, such as attention deficit hyperactivity disorder [14]. Therefore, early identification of reading difficulties may be instrumental in recognizing existing or developing mental health risks. In addition, it is widely accepted that early intervention and prevention strategies for CYP are critical: responding to early signs of distress can prevent symptoms from escalating and improve future outcomes [15]. Therefore, research into preventive mental health interventions for children and adolescents is becoming increasingly relevant and necessary [16].

Adolescents access primary care and other services for preventive health and well-being much less than other age groups [17]. As children and adolescents spend most of their day in school, schools are increasingly seen as ideal settings for delivering mental health support or interventions to young people [18-20]. The regular contact between teachers and students also means that school staff may identify those who are experiencing or at risk of developing mental health problems [21,22].

Given the low cost and scalability of digital interfaces and the widespread popularity of digital technologies among children and adolescents, digital interventions may provide new opportunities for delivering mental health support in schools [23]. Although digital support for children and adolescents has evolved and grown exponentially [24], there is a lack of research on how it can be safely and sustainably embedded in school settings. Furthermore, although the number of digital mental health apps for CYP is ever increasing, there still remains a gap in the evidence base behind them, despite their general acceptability [25], and few have been tested directly in school settings.

### Objective

This study aims to test the feasibility of embedding a low-cost, scalable, and innovative preventive digital mental health intervention in schools in the United Kingdom’s Greater Manchester area. We piloted an innovative reading ability assessment tool (Lexplore) and a web-based well-being monitoring platform (Lincus) supported by the research team, with additional training for the school staff.

## Methods

To guide our analysis regarding the sustainability and adoption of the digital reading tool and the Lincus intervention, we performed a retrospective analysis using the nonadoption, abandonment, scale up, spread, and sustainability (NASSS) framework [26]. The NASSS framework has been widely used to examine the sustainability of digital health interventions in health care settings, and its applicability in schools has been discussed elsewhere [27,28].

### Digital Components

The digital intervention comprised two components: (1) Lexplore, a reading screening assessment that uses eye-tracking technology to assess reading ability, and (2) Lincus, a digital support and well-being monitoring platform.

#### Digital Assessment: Lexplore

The Lexplore reading assessment uses eye-tracking technology to monitor a child’s eye movements while they are reading. Eye movements can provide insight into the cognitive processes behind a child’s individual reading method. Lexplore assesses reading ability (age- and sex-standardized) by calculating how long a child’s eyes fixate on words and how they move through the passage. It can examine differences in how a child’s brain processes text at lexical, syntactic, semantic, and structural levels. On the basis of this information and using machine learning, Lexplore can determine a child’s reading attainment across 5 standardized levels ranging from low to high and can highlight with precision (and often before the child, teacher, or parent has noticed) the pupils who are experiencing reading difficulties. Lexplore supports the teachers’ professional judgment and can also identify those individuals struggling with reading about whom the school or parents were unaware, often as children develop a set of coping strategies to manage their difficulties. Lexplore has recently been rolled out nationally across Swedish primary schools and is being increasingly used throughout the United Kingdom [29,30].

#### Digital Support: Lincus

Lincus is a Conformitè Europëenne marked, class 1 medical device and health and social care management platform that measures well-being across 3 domains: emotional, social, and physical. It has been implemented in several health and social care organizations, demonstrating utility in populations including individuals with long-term conditions, learning disabilities, homelessness, and multiple complex needs [31-35]. Previous research has demonstrated benefits such as improvements in reported mental health and general well-being, increased activity and perceived control of life, and better engagement and communication between health care professionals and service users [36]. Furthermore, Lincus is a configurable and customizable tool, and, specifically for this project, it was populated with child- and parent-relevant content (adapted to a simple child-friendly monitoring tool with animations for the sliding feedback scale).

### School Recruitment

The selection of schools used an opportunistic sampling method based on the networks associated with the research unit leading the study. A total of 2 schools were selected from our established links with the Manchester Healthy Schools program in Greater Manchester. Both schools were selected based on their awarded *gold status*, demonstrating their commitment to health promotion work [37].

### Participant Recruitment

All children were eligible for the study. Notably, we did not exclude children with low reading ability, as we aimed to build a digital framework that is accessible to all children. The Lincus platform is animated and image based to ensure that it is accessible to children who lack good reading skills. The research team visited the schools to provide an overview of the project to key senior staff. As they had all of the children’s relevant contact details, the schools made the initial contact with the families and children by sending correspondence from the research team to the parents or guardians. The correspondence included a letter to the parent or guardian, parental or guardian information leaflet, child information leaflet, parental or guardian consent form, and an example child assent form. Parents or guardians were informed about the project by an invitation letter and information sheet for parents or guardians. The information sheet detailed the nature and objectives of the study and possible risks associated with participation. If parents or guardians were happy with all the arrangements and for themselves and their child to take part, they were then asked to complete the parental or guardian’s informed consent form and return it to the schools.

### Ethics

The study received ethical approval from the University of Manchester Research Ethics Committee (2019-7489-11848).

### Procedure

The digital framework had two components: Lexplore initial assessment and Lincus*.*

#### Lexplore Initial Assessment

Participants initially completed a short reading ability task using the Lexplore digital platform, which involved children reading text from a computer screen with an eye-tracking sensor attached to it. On average, it took approximately between 2 and 5 minutes to complete the task. The teachers were provided with training by the Lexplore staff to enable them to perform this assessment test. After 6 weeks, participants were asked to complete the Lexplore reading ability task again.

#### Lincus

The Lincus platform was customized for child use and populated with Greater Manchester local health and well-being resources and links. Each participant was provided their own secure username, which corresponded to their participant ID number and password. This was also shared with their parents or guardians to enable them to have the option to use Lincus outside of school hours should they wish to. The Lincus platform was accessible via a web browser on a computer or tablet. Teachers were also provided with a secure username and password to have access to pseudonymized data on the platform and were provided with the appropriate training to be able to use Lincus.

Participants were asked to spend approximately 5 minutes during their free time in the morning each day for 6 weeks and complete 2 self-report surveys (lifestyle support and well-being) on the Lincus platform. Participants were encouraged to complete the surveys independently; however, the teachers were able to assist if required. The platform also included options to record observations and web-based support links for children, parents or guardians, and teachers.

Following the intervention and follow-up assessment, children, their parents or guardians, and their teachers were invited to a qualitative focus group or workshop to provide feedback on the digital framework.

### Data Collection

Data were collected via the Lexplore and Lincus platforms separately. The data from Lexplore included a score of reading age, ability, comprehension, and speed for each participant during the testing and follow-up phases of the project. The Lincus platform collected self-report data on emotional, social, and physical well-being. Lincus data also included usability and engagement metrics (ie, how many times the child used the platform, modules accessed, and duration of use). If a participant withdrew during the project, no further data were collected from them; however, historic data were retained. Qualitative feedback from parents was collected via their evening appointments rather than workshops, as the school advised this would be the most suitable way to collect parent feedback. Feedback from the teaching staff was also obtained during the parents’ evening appointments because of the limited capacity and time for conducting a separate focus group or workshop. Feedback from children was gathered as part of a group workshop.

### Data Analysis

We used the NASSS framework [26] as a post hoc method to analyze the data and understand the barriers and facilitators to implementing the intervention (Textbox 1).

**The nonadoption, abandonment, scale up, spread, and sustainability framework.**

**Condition**

- The prevalence of poor mental health in children and adolescents has significantly increased in the United Kingdom and, in recent years, has become a public health concern worldwide, with Greater Manchester being a particular area of high need.

**Technology**

- An integrated digital framework where both had good usability, and Lincus had been co-designed and tailored for the Greater Manchester population. Both were standalone systems outside the usual technologies used in schools.
- Data made available were reported as clear and helpful; however, engagement from teaching assistants and parents was poor. Therefore, education about how to access the data was found wanting.
- Minor issues were experienced with access to Lincus, such as log-in difficulties and problems with firewall settings. These difficulties were sometimes readily solved or would have been relatively easy to solve with appropriate communication from the staff.
- The technology was supplied through project grant funding; however, both were low-cost technologies. Lexplore has already been extended and deployed at other schools.

**Value proposition**

There was evidence of both demand-side and supply-side value:

- To secure funding for the project, a clear business case was presented for the Lincus system and Lexplore assessment tool (supply-side value).
- The desirability (demand-side value) existed, with enthusiasm from senior staff regarding the technologies being low cost and scalable, and innovative digital screening platforms to identify and support children who were most vulnerable to mental health problems.
- However, some parents did not perceive the digital system to have value, as they did not consider their children to be needing mental health support.

**Adopter system**

- The staff found the disruption of their morning routine as adding to pressures and demands.
- Teaching assistants are responsible for a large number of children and tasks and can perceive additional responsibilities as burdensome.

**The organization**

- Staff reported that existing pressures limited their ability to engage fully with the project.
- Recruitment and consent process was deemed time consuming.

**Wider system**

- Children and young people’s mental health and well-being is and continues to be a key priority; Greater Manchester is, in particular, an area of high unmet need.
- Since the COVID-19 pandemic, digital technologies are increasingly being used to support children and young people within educational and health care settings.

**Embedding and adaptation over time**

- There is scope for adaptation over time of both the system and in the way the technology is deployed.
- This study found a lack of organizational resilience to changes and the embedding of new technologies because of staff capacity and workload.

## Results

### Overview

The project was aimed at children aged between 6 and 10 years. The first school completed testing in January 2020, with 34 children and 5 teachers across year 4 or 3rd grade and year 5 or 4th grade (mean age 9 years; 19/34, 56% females, 15/34, 44% males). The uptake was 29.6% (34/115; total number of children approached being 115 across the 4 classes). The second school agreed to participate from the year 4 or 3rd grade and year 5 or 4th grade, and their consent forms were completed. However, staffing difficulties and sicknesses meant they had to withdraw.

A total of 4 more schools were contacted across Greater Manchester. A total of 2 schools did not respond and 2 schools expressed interest; however, 1 could not confirm participation because they were waiting for a new head teacher to commence. The other later declined, citing that the school was extremely busy with other demands. Access to further schools became impossible following the COVID-19 lockdown in the United Kingdom from March 23, 2020.

### User Engagement

During the 6-week period of data collection, Lexplore assessments were conducted twice in each school. All children completed an initial assessment, and 88% (30/34) completed their follow-up reading assessments. The follow-up consent forms were not returned by parents for the second round of assessment; these children were not tested at follow-up. A total of 322 Lincus well-being surveys were recorded. On average, 46 well-being surveys were recorded per week, equating to approximately 1 well-being survey per user per week in total (6 per user per month). Most children were classified by Lincus as *regular* (≥1 per week) survey users. There was a significant drop in use over the 2-week Christmas holidays, although 6% (2/34) of children reported logging in at home; this can explain the lower average during December.

Approximately 18% (4/34) of children did not complete any well-being surveys; the reasons provided were that they had never logged in because of late attendance, sickness, or not having time in the morning to complete the survey tasks.

### Poststudy Consultations With the Adopters (the Adopter System—NASSS Framework)

#### Children

Approximately 68% (23/34) of children participated in group feedback (14/23, 61% from year 4 or 3rd grade and 9/34, 39% from year 5 or 4th grade). Overall, there was a favorable opinion among the children regarding the digital platforms, as indicated by their quotes (Textbox 2).

**Quotes from children describing how they felt about Lexplore, Lincus, and confidentiality.**

- “Reading was fun. It was futuristic”; “It picked up I have a lazy eye”; “it’s cool!”
- “It felt good and weird to tell the internet how I feel”; “You can tell the truth about yourself instead of telling the whole world”; “Sometimes I don’t want to say how I’m feeling so instead of talking, I liked putting down and people can read it.”
- “I didn’t answer the truth on sleep in case I got into trouble for staying up and being tired in the classroom the next day so said I slept better.”
- “I lied about my social life and put the score where I wanted it to be and not where it was.”
- “Anxiety—I put it lower I was embarrassed, I didn’t want to score it in case anyone found out, my friend had anxiety and they got bullied so didn’t want the same to happen to me.”

Qualitative feedback demonstrated that the Lincus content was mostly found acceptable and relevant by users, although some domains of well-being needed explanation by the teaching assistant (TA; eg, control of life and appetite). Children reported that the pictures helped them understand the scale. Completion time of the measures was found to be acceptable for children reporting quick response times. They described finding it easy to complete the scales, and once they were able to access the platform following log-in, they required no further support.

The request for daily log-ins to Lincus was deemed burdensome by some children, with suggestions of a couple of times a week being preferable. Log-in difficulties were highlighted, resulting in children being locked out of their accounts and, consequently, being unable to complete the survey during their allocated time. In addition, some children reported that having to engage with the platforms meant that they missed out on other enjoyable activities alongside their nonparticipating peers.

Some children expressed concerns regarding the confidentiality of their Lincus data; specifically, they were worried that their teachers, parents, and other third parties might have access to the data, which may have influenced their responses. Moreover, some children suggested that they inflated their scores to be more desirable (Textbox 2). The duration of the Lexplore assessment was approximately 5 minutes; several children commented that they would have liked the process to be even quicker. However, 2 sessions were acceptable to them.

#### Parents

The school suggested that we gather feedback at the parents’ evening appointments to maximize parental engagement. The parents of 21% (7/34) of children were interviewed regarding the study. None of these parents had accessed their child’s well-being survey, citing reasons such as being too busy, having no concern regarding their child’s well-being, and log-in difficulties. Furthermore, parents reported that they were not aware of the health and well-being resources held on Lincus. At the time of the interview, parents had not yet received any feedback from teachers regarding their child’s Lexplore results, despite this being part of the teachers’ training by research staff at the beginning of the program.

#### Teachers

Out of 4 teachers across the year groups, 2 (50%) provided feedback at the parents’ evening appointment (one year 4 or 3rd grade and one year 5 or 4th grade teacher). The year 4 or 3rd grade teacher rated both platforms positively overall. However, they had forgotten that Lincus hosted a wealth of mental health and well-being resources despite receiving training on the platform. The staff were provided with personal log-in details; however, none of them had accessed the platform. The year 5 or 4th grade teacher felt less informed, as they did not attend the training session and, therefore, were not aware of the potential value and capabilities of the Lincus platform. They suggested that rather than having to log on to each child’s profile regularly, which was considered by some as too time consuming, receiving notifications regarding a child who was scoring consistently low would be more beneficial.

The Lexplore results were reported to be consistent with their own assessment of the children’s reading performance; however, they liked that it added an objective measure. They believed these scores could be helpful in following up on children who were struggling and aid in discussion with their parents. The teachers reflected that if everyone in the class had opted in, the completion of the assessments would have been less disruptive, as they would have been able to complete all assessments together in 1 classroom. However, they also felt that it would not have been feasible because of the limited staff capacity to monitor and support all the children completing the assessment.

#### Teaching Staff

Both TAs were solely responsible for supporting the children in using the Lincus survey and completing their reading assessments. Feedback was based on 1 TA who worked across the two year 4 or 3rd grade classes and was available at the time of the consultation. This consultation identified the implementation strategies, barriers, and facilitators.

### Implementation Strategies

TAs were asked to support the daily input of Lincus well-being data as per the study protocol. However, as the study progressed, the TAs reported that the children became more self-directed and required fewer reminders. They were able to seek out the tablets independently. All well-being surveys were conducted before formal lessons. Additional training was provided for the TAs to complete the Lexplore reading assessments at the 2 assessment points. Each class was allocated an assessment period, which took approximately half a day to complete.

### Implementation Barriers and Facilitators

Log-in difficulties were a barrier that was identified early on in the intervention, which locked some children out of completing their survey in the time allocated. Log-in information or passwords were set by the system and were written down and accessible to the children; however, because the password entry box was blinded to them, this increased the frequency of mistakes. The suggestions were that children could choose their own password or log-in information that they would be able to remember and easily type out to mitigate this. Once the children had completed a few surveys, it was evident that they could input their well-being data without further support.

Teachers reported that they found it challenging to incorporate daily well-being surveys into their demanding morning routines, although the staff did not feel that other times of the day were more suitable. Completing Lexplore was not deemed time consuming for the actual assessment. However, the organizational logistics around implementing assessments were viewed as labor intensive (eg, finding a suitable and available room, collecting and preparing children for the assessment, and then returning them to classrooms). This was perceived negatively, as typical reading assessments are spaced out and do not require equipment or extra organization.

The availability of digital equipment (tablets) across classes at the same time was highlighted as a challenge. Log-in difficulties that timed the children out of the well-being surveys reduced user engagement. Poor parental engagement in providing consent was an additional barrier in the recruitment context, resulting in a delayed start of the study. This meant that there was a 3-month interval between staff training on the digital platforms and the study’s commencement, leading to the staff feeling less familiar with the platform and its operation.

No difficulties were explicitly reported in maintaining the children’s engagement as a barrier to implementation. Parental involvement was not a necessity for the project once it had started, clearly demonstrated by the high user engagement of children in the school setting.

## Discussion

### Principal Findings

#### Overview

The primary aim of this project was to examine the feasibility and acceptability of embedding a low-cost, scalable, and innovative assessment and preventive digital mental health tool for schoolchildren in the Greater Manchester area. Overall, children found this digital intervention engaging, usable, and acceptable. However, despite widespread enthusiasm and recognition of the potential added value from head teachers during the consultation phases of the project, we met significant implementation barriers. Consistent with the findings of Edridge et al [27,28], all 6 themes representing implementation barriers within the NASSS framework emerged: technology, value proposition, the adopter system, the organization, wider system, and embedding and adaptation over time.

#### Technology

Minor issues were experienced with log-in access to Lincus. These difficulties were sometimes readily solved or would have been relatively easy to solve with appropriate communication from the staff. Lack of communication by the staff resulted in some difficulties remaining unresolved. This then became a more significant barrier and prevented or reduced user engagement. Difficulties in accessing Lincus could be readily mitigated by providing personal log-in details. Additional barriers were identified by the second school (which later withdrew), with a security firewall preventing access to the platform. This is a straightforward problem to resolve from a technical perspective by enabling access to the platform through the firewall. However, it presented a significant implementation barrier, as the teaching staff did not readily know how to identify, report, or resolve the access problem. These are important considerations for future work across educational settings, as such barriers may feel overwhelming or burdensome to staff already under significant pressure and may influence their participation.

#### Value Proposition

There was evidence of both demand-side and supply-side value. To secure funding for the project, a clear business case was presented for the Lincus system and the Lexplore assessment tool (supply-side value). The desirability (demand-side value) existed, with enthusiasm from senior staff regarding the technologies representing low-cost, scalable, and innovative digital screening platforms for the early identification of and support for children most vulnerable to mental health problems. However, the benefits could have been reinforced with the TA staff to improve motivation to engage with the project. In addition, some parents did not perceive the digital system as having value as they did not believe their children needed mental health support.

#### The Adopter System

Overall, children reported finding both Lexplore and Lincus acceptable, and their feedback suggested that they would continue to engage with both platforms. However, barriers were identified in engagement for the staff and parents. For instance, participation required some change to the morning routine for teaching staff, which added strain to an already demanding schedule. TAs bear the weight of many responsibilities for a large number of children and, therefore, may be at risk of perceiving additional tasks as burdensome. In our study, we found that TA availability and willingness are integral to the feasibility of school-based mental health programs. Therefore, recruiting TAs who support the aims and have a clear understanding of the program benefits is key to success. Ensuring that TAs have sufficient time to support children among their other daily tasks will also be an important element of future implementation. Enthusiasm from head teachers would have to be met by their leadership in championing the assessment tools and supporting the teachers and TAs in delivering the program. Although parents could be categorized as being part of the adopter system, their role was not required for children’s successful engagement with the platforms. Feedback from the interviewed parents was that they were not concerned regarding their children’s mental health and well-being. This could explain why they did not access the platforms themselves, as they did not consider their children to have any mental health–related concerns and, therefore, felt there was no need to check up on them. As outlined above, previous work has highlighted the concerns of parents and schools regarding children and adolescent mental health. However, the sample we were able to recruit for this feasibility pilot may not be representative of the parents we originally aimed to target, as this study’s parents did not report having worries or concerns regarding their children. An explanation might be that families from more socioeconomically deprived or ethnically diverse backgrounds are less likely to take part in research so that children who may be more likely to experience mental health problems are less likely to engage in research and are underrepresented in the samples [38]. Future research should consider the barriers to participation from underserved groups; this is increasingly important to ensure that samples are inclusive and involve those with unmet needs and who may be most likely to benefit from these interventions.

#### The Organization

The staff reported that significant pressures on them, though unrelated to this study, limited their ability to engage fully with the project. The consenting and recruitment process for children was time consuming and required multiple periods of engagement with the school staff, resulting in relatively low rates of consent. Successful roll out of digital platforms for future routine use in schools is likely to need more extensive engagement with teachers and TAs. In our view, the organizational barriers we identified are largely surmountable, some more readily than others. We recommend future research projects to ensure adequate upfront engagement with schools so that all school staff, including senior leadership, frontline teaching staff, and TAs, are fully supported to deliver the research.

#### Wider System

The wider context was and continues to be supportive of mental health and well-being within schools. Particularly since the COVID-19 pandemic, digital technologies are increasingly being deployed to support CYP within schools and across mental health services [25]. CYP's mental health and well-being are key priorities and continue to be so. Greater Manchester is a particular area of high unmet need for CYP mental health [3]. Not feeling listened to and perceived social stigma have previously been identified as key barriers to CYPs engaging with specialist services and seeking help [39]. Personalized digital tools such as Lexplore and Lincus allow CYP to have their voices heard in a safe and nonstigmatized way, in contrast to meeting school nurses or counselors or their general practitioners with their parents.

#### Embedding and Adaptation Over Time

There is scope for adaptation over time of both the systems and in the way the technology is deployed. However, we found a lack of organizational resilience regarding changes and the embedding of new technologies in this study because of limited staff capacity and a focus on other school priorities.

### Strengths, Limitations, and Recommendations

There are several strengths to this feasibility study. We used an established digital health intervention framework to evaluate the adoption, scale up, spread, and sustainability, and in the school where engagement was possible, we recruited nearly one-third of the eligible children in a relatively short space of time. The Lincus platform was easily adaptable to embed local information that was relevant and contextualized to schools and children. Support from school leadership highlighted the need for such digital programs to supplement ongoing mental health delivery in schools. Preventive strategies are key in early identification to provide timely support to reduce the risk of development or escalation of mental health problems. Both the Lexplore and Lincus interventions may be beneficial in enabling the staff and parents to identify issues by monitoring reading ability and well-being. The feasibility study demonstrated the acceptability of the digital intervention to staff and children and the willingness of children and parents to consent to engage with digital tools.

This study also has some important limitations. We do not have information regarding children who did not participate but were eligible to do so. This makes the assessment of bias and representativeness of the sample unclear. Demographic data, such as ethnicity and socioeconomic status were not captured. The overall recruitment of children was limited because of the challenges in engaging key school staff and parents. Furthermore, the intervention was only tested in 1 region of the country with opportunistic sampling and, therefore, does not indicate how this would work in other settings across the United Kingdom. Although the qualitative data show a positive appraisal of both platforms, and children generally had a positive experience using the tools, this may not have been the case for all children who were eligible to participate. Similarly, we had limited participation from staff and parents, making it difficult to determine the acceptability of the digital tools for parents, carers, and teachers. This may not be a problem in the future if children accept and adopt the tools and parents and teachers follow their lead. Another key consideration is funding and resource availability: the success of both implementing and adopting digital technologies in a school setting means all children can access the equipment they require to participate, such as tablets, laptops, and computers. This might mean additional support and resources for children from lower socioeconomic backgrounds. We conducted this as a *standalone* program; it may work better when integrated as part of personal, social, health, and economic education or other extant modules within the school curriculum. Finally, it was not specifically linked to mental health programs in schools; this is an important consideration if the platforms are to become successfully embedded in schools. Using the platform as an educational tool or integrating it into other digital educational tools as part of the curriculum would guarantee adoption with added health benefits. Embedding digital health interventions within the existing school structure and programs may be a better way to increase involvement and commitment from staff, parents, and children.

### Future Work

The daily demands faced by the teaching staff are key barriers to embedding any new technology requiring significant teacher input in schools. Despite the senior staff’s willingness to welcome the new technology and a desire from some teachers and TAs to take part in the research (which they saw as relevant to their practice), much more work is needed to demonstrate the value of digital platforms to staff and parents. Future deployments should work closely with schools, children, and parents from the outset to codevelop the platform with them to respond to their needs and facilitate adaptation over time, as opposed to implementing ready-made tools. Identifying research champions in classes or year groups could offer a route to more seamless engagement [40]. This suggests that future studies could bypass the need for ongoing TA involvement, and this may also be likely to encourage participation from other children.

The COVID-19 pandemic has created far greater digital engagement from schools, teachers, pupils, and parents. This may present an opportunity for teaching staff to be able to engage more readily in the future with web-based platforms such as Lincus and Lexplore.

### Conclusions

The key aim of this study was to embed a low-cost, scalable, and innovative digital mental health intervention in schools in the Greater Manchester area to identify and provide timely support for children most at risk of mental health problems. Overall, the digital platforms were well-received, and the study revealed important barriers and facilitators that can provide key information and associated recommendations for conducting future research in this setting. The landscape has changed dramatically during the COVID-19 pandemic, with a spike in interest in the use of digital technologies to manage health and well-being. Some of the difficulties we encountered in the feasibility of widening the implementation of digital mental health and educational support tools may have now been mitigated. Where staff felt adequately supported, both platforms could be delivered feasibly, and overall, children and parents found them acceptable. However, the teaching staff play an instrumental role in the success of implementing digital technologies, and staff attitudes influence the degree to which new technologies are accepted within traditional working practices. Therefore, future options should minimize the need for staff and parent involvement and focus on widening children’s participation. Furthermore, staff training, funding, resources, and staff willingness to engage and participate must all be considered for the successful implementation of digital mental health solutions in schools. Although this study did encounter some difficulties, it provided interesting pilot data that highlight the potential benefits of implementing digital health tools within a school setting.

## Abbreviations

CYP: children and young people
NASSS: nonadoption, abandonment, scale up, spread, and sustainability
TA: teaching assistant
